# 969 Development of a Pre-Operative Burn Risk Level Assessment Tool

**DOI:** 10.1093/jbcr/iraf019.500

**Published:** 2025-04-01

**Authors:** Monica Hutson, Jason Sheaffer, Carlos Jimenez, Alexis McQuitty, Steven Wolf

**Affiliations:** University of Texas Medical Branch, Blocker Burn Unit; University of Texas Medical Branch, Blocker Burn Unit; University of Texas Medical Branch, Blocker Burn Unit; University of Texas Medical Branch, Blocker Burn Unit; University of Texas Medical Branch, Blocker Burn Unit

## Abstract

**Introduction:**

Preoperative assessments typically center on ensuring patient safety, surgical readiness, and evaluating medical history alongside potential anesthesia and surgical risks. Burn severity profoundly influences surgical timing, complexity, anesthesia considerations, and postoperative care needs. Post-operative complications among burn patients include hypothermia, acute kidney injury (AKI), and cardiac arrest related to hypovolemic shock. This improvement aims to align anesthesia staffing and postoperative care allocation to optimize burn patient outcomes through the development of a Pre-Operative Burn Risk Level Assessment Tool.

**Methods:**

Risk levels were determined by inpatient acuity considerations, Total Body Surface Area (TBSA) burn, and abnormal findings. Low risk: routine burn operation, TBSA ≤ 20%, or no abnormal findings. Moderate risk: first burn operation, past perioperative adverse event, TBSA 20-40%, or stabilized abnormal findings. High risk: operation ≤ 48 hours post-burn, TBSA > 40%, or unstabilized abnormal findings. Anesthesia staffing: low risk – standard; moderate risk – one faculty anesthesiologist to two patients, one mid-level resident to one patient; high risk – one faculty anesthesiologist to one patient, one upper-level resident to one patient. Post-operative disposition: low risk – PACU; moderate risk – ICU or PACU based on intra-operative response; high risk – ICU.

**Results:**

A total of 63 surgical cases were reviewed over the course of 6 months. Risk level underscoring occurred in 5 surgical cases, though patient disposition and anesthesia staffing were maintained at the higher level. Inappropriate staffing and patient disposition discrepancies relative to risk level each occurred separately in 1 surgical case. All surgical cases were executed without untoward incidents.

**Conclusions:**

Risk level determination and communication were integrated into the electronic surgical posting process. This initiative necessitated adjustments in anesthesia faculty ratios and assignments based on resident experience. Moderate risk level criteria slightly increased the number of patients returning to the ICU for post-operative care. Both departments effectively managed changes without increasing their staffing levels. Implementation of the Pre-Operative Burn Risk Level Assessment Tool has led to a decrease in post-operative safety events among burn patients.

**Applicability of Research to Practice:**

A quality improvement project to reduce the occurrence of post-operative burn patient safety events by designating and communicating a pre-operative burn risk level.

**Funding for the Study:**

N/A

